# 132. Evaluation Phage Cocktails in Combination with Ciprofloxacin Against Multidrug-Resistant *Pseudomonas aeruginosa* Overexpressing MexAB-OprM Efflux Systems

**DOI:** 10.1093/ofid/ofab466.132

**Published:** 2021-12-04

**Authors:** Dana Holger, Katherine Lev, Natasha Bhutani, Razieh Kebriaei, Taylor Morrisette, Susan Lehman, Jose Alexander, Michael J Rybak

**Affiliations:** 1 Anti-Infective Research Laboratory, Department of Pharmacy Practice, Eugene Applebaum College of Pharmacy and Health Sciences, Wayne State University, Detroit, Michigan, detroit, Michigan; 2 Wayne State University, Detroit, Michigan; 3 Center for Biologics Evaluation and Research, U.S. Food and Drug Administration, Silver Spring, Maryland, USA, Silver Spring, Maryland; 4 AdventHealth Orlando, Orlando, FL; 5 Wayne State University / Detroit Medical Center, Detroit, Michigan

## Abstract

**Background:**

Multidrug-resistant (MDR) *Pseudomonas aeruginosa* infections are increasing in prevalence and cause significant mortality. The MexAB-OprM efflux system confers resistance to a wide range of drugs, including β-lactams, fluoroquinolones, tetracyclines, and macrolides. Obligately lytic bacteriophages (phages) are viruses that infect and kill bacteria. Phage therapy has been suggested as an alternative treatment option in combination with traditional antibiotics. The objective of this study was to determine the ability of a phage cocktail in combination with ciprofloxacin (CIP) to improve bacterial killing and/or prevent the emergence of phage resistance in MDR *P. aeruginosa*.

**Methods:**

Initial bacterial susceptibility to phage was evaluated with three newly isolated phages (phages EM, LL, and A6) against ten clinical MDR *P. aeruginosa* isolates. Theoretical multiplicity of infection (tMOI) optimization was performed with two phages with the broadest initial susceptibility (tMOI: 1.0 chosen for further analysis). A preliminary evaluation was performed with *P. aeruginosa* R9316 (carbapenem-resistant clinical strain with MexAB-OprM overexpression, as determined previously by quantitative real-time PCR). Synergy for phage cocktail combinations (≥ 2-log_10_ CFU/mL kill compared to most effective single agent at 24 h), bactericidal activity for all samples (≥ 3-log_10_ CFU/mL reduction at 24 h compared to starting inoculum), and phage resistance development were evaluated in time kill analyses (TKA).

**Results:**

R9316 is a MDR *P. aeruginosa* isolate with a CIP MIC of 2 mg/L. Phage cocktails as monotherapy had little impact on bacterial eradication (reduction: 1.19 log_10_ CFU/mL). However, the addition of CIP to phage cocktails of EM and LL phages led to synergistic and bactericidal effects (reduction: 3.92 log_10_ CFU/mL). Furthermore, phage resistance was observed in the phage monotherapy regimens. Whereas the addition of CIP was shown to prevent the emergence of phage resistance in some regimens.

**Conclusion:**

Our results show synergistic activity and prevention of phage resistance with phage cocktail-antibiotic combinations against MDR *P. aeruginosa.* Further research is needed to determine the impact of phage cocktail therapy on additional strains and clinical outcomes.

**Disclosures:**

**Michael J. Rybak, PharmD, MPH, PhD**, **Paratek Pharmaceuticals** (Research Grant or Support)

